# Visual Performance in Patients with Neovascular Age-Related Macular Degeneration Undergoing Treatment with Intravitreal Ranibizumab

**DOI:** 10.1155/2013/268438

**Published:** 2013-02-21

**Authors:** Sarah Sabour-Pickett, James Loughman, John M. Nolan, Jim Stack, Konrad Pesudovs, Katherine A. Meagher, Stephen Beatty

**Affiliations:** ^1^Department of Optometry, School of Physics, Dublin Institute of Technology, Dublin, Ireland; ^2^Institute of Eye Surgery and Institute of Vision Research, Whitfield Clinic, Waterford, Ireland; ^3^Macular Pigment Research Group, Waterford Institute of Technology, Waterford, Ireland; ^4^African Vision Research Institute, Faculty of Health Sciences, University of KwaZulu-Natal, Durban, South Africa; ^5^NH & MRC Centre for Clinical Eye Research, Department of Optometry and Vision Science, Flinders Medical Centre & Flinders University of South Australia, Bedford Park, Australia

## Abstract

*Purpose*. To assess visual function and its response to serial intravitreal ranibizumab (Lucentis, Genentech) in patients with neovascular age-related macular degeneration (nv-AMD). *Methods*. Forty-seven eyes of 47 patients with nv-AMD, and corrected distance visual acuity (CDVA) logMAR 0.7 or better, undergoing intravitreal injections of ranibizumab, were enrolled into this prospective study. Visual function was assessed using a range of psychophysical tests, while mean foveal thickness (MFT) was determined by optical coherence tomography (OCT). *Results*. Group mean (±sd) MFT reduced significantly from baseline (233 (±59)) to exit (205 (±40)) (*P* = 0.001). CDVA exhibited no change between baseline and exit visits (*P* = 0.48 and *P* = 0.31, resp.). Measures of visual function that did exhibit statistically significant improvements (*P* < 0.05 for all) included reading acuity, reading speed, mesopic and photopic contrast sensitivity (CS), mesopic and photopic glare disability (GD), and retinotopic ocular sensitivity (ROS) at all eccentricities. *Conclusion*. Eyes with nv-AMD undergoing intravitreal ranibizumab injections exhibit improvements in many parameters of visual function. Outcome measures other than CDVA, such as CS, GD, and ROS, should not only be considered in the design of studies investigating nv-AMD, but also in treatment and retreatment strategies for patients with the condition.

## 1. Introduction

Choroidal neovascularisation (CNV), a manifestation of neovascular age-related macular degeneration (nv-AMD), is characterised by the growth of abnormal choroidal blood vessels, which penetrate Bruch's membrane and sometimes the retinal pigment epithelium (RPE) [1]. If left untreated, the leakage results in subretinal and/or retinal scarring, with consequential and irreversible loss of central vision [2].

Vascular endothelial growth factor (VEGF), a diffusible cytokine, is an important factor promoting CNV in AMD [3, 4]. Clinical trials have confirmed the efficacy of ranibizumab (Lucentis, Genentech), an inhibitor of VEGF, over laser therapy with intravenous Visudyne and over observation alone [5, 6]. 

Currently, corrected distance visual acuity (CDVA), a measure of the resolving power of the eye at high contrast [7], represents the standard vision-related outcome measure for management of nv-AMD. However, CDVA is not a true reflection of daily visual experience in a world with few visual stimuli at such high levels of contrast, suggesting that perhaps other measures of visual function may be more appropriate in assessing visual performance and experience in patients with nv-AMD [8, 9]. 

Techniques alternative to CDVA, and relevant to nv-AMD, include (but are not restricted to): contrast sensitivity (CS) [10, 11]; glare disability (GD) [12]; retinotopic ocular sensitivity (ROS) [13–16]; preferential hyperacuity [17]; reading performance [18–20]; and subject-reported experience by validated questionnaire [21–23].

In this study, we investigated visual performance, and its response to treatment, in patients with nv-AMD undergoing treatment with intravitreal ranibizumab, and with baseline CDVA of logMAR 0.7 or better. Considering that baseline CDVA levels have been shown to influence the extent to which CDVA improves following treatment [24, 25], we sought to explore the impact of treatment on visual function in this subset of subjects and investigate if there are observable improvements/changes when psychophysical parameters other than CDVA are employed as outcome measures.

## 2. Methods 

### 2.1. Patients

Suitability for inclusion in the study was confirmed by an ophthalmologist, in compliance with the following inclusion criteria: the study eye must be suffering from active nv-AMD (fluid and/or cysts in the retina resulting from blood vessels that are actively leaking, as observed on fundus fluorescein angiography (FFA)) and be scheduled to commence, recommence, or continue a course of intravitreal ranibizumab; have a baseline CDVA of logMAR 0.7 or better; and exhibit no visually important ocular comorbidity. All patients were recruited from the Institute of Eye Surgery, Whitfield Clinic, Waterford. Exclusion criteria included a history of diabetes mellitus. In cases where both eyes were being treated, the eye with the better CDVA was selected for the study. Ethics approval was granted by the Dublin Institute of Techonology Ethics Committee, and informed consent was secured from each subject. The research was conducted in accordance with the principles of the Declaration of Helsinki. 

 Visual function and optical coherence tomography (OCT) data were collected at baseline, and at monthly intervals (midway between monthly ranibizumab injections) within the 12 month study period. For the purposes of statistical analysis, baseline and exit study visit data were used. An exit visit was defined as the patient's final study visit (two weeks after the preceding and final intravitreal injection in the study). Subjects exited the study either when the study period came to an end (*n* = 20; after a maximum follow-up of 11 months; some of these patients may have continued with further intravitreal injections of ranibizumab following closure of the study), when treatment was discontinued on clinical grounds (*n* = 23; in these cases it was deemed, clinically, that maximum realisable benefits of treatment had been acheived), when the patient was unable to continue in the study for unrelated health reasons (*n* = 2), or when the patient elected to discontinue his/her participation in the study (*n* = 2).

 A diagnosis of nv-AMD was made by a retinal specialist on the basis of clinical examination, OCT, and FFA. The standard regime of treatment (following initial diagnosis) included three consecutive monthly injections, followed by monthly evaluation for further treatment. Subsequent injections were administered based on signs of lesion activity on OCT and FFA. This protocol has been previously described [26], and typically upon resolution of fluid and/or cysts (determined by OCT), one more intravitreal injection of ranibizumab was administered and two weeks following that intraocular injection, FFA was repeated. Where lesion inactivity was angiographically confirmed, treatment was discontinued.

### 2.2. Visual Performance

Corrected distance visual acuity was measured for the study eye monocularly, and with the patient's best subjective refraction using the logMAR chart provided by a letter chart (Test Chart 2000 PRO:Thomson Software Solutions, Hertfordshire, England) at a testing distance of 4 m. 

CS was measured using the sine wave grating-based Functional Vision Analyser (Stereo Optical Co., Inc—Chicago, USA). Testing was performed under mesopic (3 candela per square metre [cd/m^2^]) and photopic (85 cd/m^2^) conditions. This test was repeated in a similar manner under mesopic and photopic conditions but in the presence of an inbuilt circumferential LED glare source (1 lux for mesopic and 10 lux for photopic glare testing) to assess GD [27].

ROS was measured by microperimetry (Microperimeter MP 1; Nidek Technologies Srl, Albignasego, Italy) adhering to a previously described protocol [28]. Microperimetry assesses macular function by examining the light differential threshold at specific points on the retina, under direct visualisation of the fundus. The patient was instructed to fixate a red cross spanning three degrees from fixation, for the duration of the test. The examination pattern comprised 21 stimuli, presented under mesopic background illumination of 1.27 cd/m^2^ (4 asb). The stimulus size was Goldmann III (26 minutes of arc), of white colour and of 200 msec presentation duration. Stimulus intensity ranged from 20 dB (dimmest [4 asb]) to 0 dB (brightest [400 asb]); an increase of 1 dB equates to 0.1 log reduction in stimulus intensity (asb). Thresholds were determined using a 4-2 linear staircase strategy. ROS was calculated for three areas: fixation (one stimulus); within central 5 degrees (including fixation) using an average of nine stimuli; and within 16 degrees of fixation (average of 21 stimuli).

 Reading speed and near visual acuity (LogRAD [log reading acuity]) were measured with an English version of the standardised Radner reading chart adhering to a previously described protocol [29]. 

Every effort was made to minimise any learning effects that may potentially influence psychophysical outcomes, which included: careful explanation of each task, demonstrations, test cards and/or trial runs where possible (at baseline), and the exclusion of any unreliable data.

### 2.3. Assessment of Retinal Thickness

Optical coherence tomography (OCT) was performed using a Topcon 3D OCT-1000 (version 3.01, Mark I; Topcon Corporation, Tokyo, Japan). The central 1 mm mean foveal thickness (MFT) was obtained from typical EDTRS (Early Treatment Diabetic Retinopathy Study) macular thickness maps [30]. The central foveal thickness was defined as the distance between the inner and outer boundaries of the scanned image, identified using a validated internal algorithm, and did not include any fluid under the RPE. Mean foveal volume (MFV) was determined in a similar fashion.

### 2.4. Statistical Analysis

Descriptive statistics were calculated for all measured variables, including demographic, ocular, psychophysical, and morphological data. Visual acuity rating scores [31] were used for the statistical analysis of CDVA data. Statistical analysis was performed using the software package PASW Statistics 18.0 (IBM Corp., Somers, NY, USA).

Baseline and exit visit measures were compared using the paired-samples *t*-test. Correlations between observed changes in MFT (and MFV) and observed changes in psychophysical measures (between baseline and exit study visits) following serial anti-VEGF therapy were investigated using Pearson correlations. Power analysis, for the sample size of 43 subjects (following dropouts), yielded the following results: for detecting a correlation of 0.5, the power of a sample of this size is 0.94; for detecting a change of half a standard deviation on a paired *t* test, the power is 0.89. Tests were 2-sided in all analyses and the 5% level of significance was used throughout, without adjustment for multiple tests. We were not in favour of Bonferroni adjustment as we felt it was too severe and may result in failure to reject null hypotheses where rejection is warranted (i.e., it leads to increased likelihood of Type II statistical errors).

## 3. Results

Forty-seven patients (47 study eyes) met the inclusion criteria and were recruited into this study. Of these, 31 were already undergoing treatment when recruited (mean (±sd) and range of duration of prior treatment: 7 (±5) and 1–20 months, resp.). Eight of the 47 study patients were concurrently undergoing serial intravitreal ranibizumab treatment in their fellow eye, at enrolment. Beyond baseline, 43 patients continued in the study, and the mean (±sd) number of visits for these subjects was 6 (±2.6), with a range of 2–10 study visits (three subjects had only two study visits). A total of 248 injections of ranibizumab were administered to the study eyes over the course of the investigation. The mean (±sd) and range of the number of injections per patient was 5.4 (±2.8) and 1–10, respectively, over the course of the study. 

Baseline measurements were obtained 1-2 days prior to the first injections (of that course of injections) in the 16 participants who were commencing or recommencing treatment, one of whom did not continue beyond baseline. This subgroup (*n* = 15) will be henceforth termed “Subgroup A”. We anticipated that data in Subgroup A (as a result of the recent (re)activation of nv-AMD in this subgroup), might differ from study eyes where serial intravitreal treatment was already underway, particularly since it has been shown that the greatest improvements in vision are typically obtained in the first three months of treatment [25, 32] and therefore warranted separate analysis.

 The results of tests to investigate which of the measured parameters exhibited significant change over the course of the study period are reported in Table 1. Of note, there was no statistically significant change in CDVA for the study group (*P* = 0.480), or Subgroup A (*P* = 0.387), between baseline and exit.

 Relationships between observed changes in MFT and observed changes in parameters of visual function for the study group and Subgroup A are displayed in Table 2.

## 4. Discussion 

The purpose of this study was to evaluate outcome measures for patients with nv-AMD undergoing intravitreal ranibizumab therapy. Over the course of the study, MFT and MFV decreased in response to treatment, consistent with previous studies [26, 33, 34]. Parameters of visual function that improved for the study group and Subgroup A included reading speed (at best baseline LogRad value), mean reading speed, CS under mesopic conditions at low and high spatial frequencies, CS under photopic conditions at high spatial frequencies, GD under mesopic conditions for low and high spatial frequencies, GD under photopic conditions at low and high spatial frequencies, and ROS within the central 5 and central 16 degrees of fixation.

 Mean CDVA did not improve significantly over the course of the investigation for the study group or Subgroup A. We believe this finding is attributable to our inclusion criteria and to the short period of follow-up in this study. Other studies have shown that poor baseline CDVA is associated with a greater benefit of treatment in terms of this outcome measure [24, 25], and this observation is consistent with the findings of Williams and Blyth, who reported no significant improvement in CDVA in patients with nv-AMD in patients where baseline CDVA was 6/12 (logMAR 0.3) or better [35]. Given that, in the current study, all study eyes had baseline CDVA of logMAR 0.7 or better (indeed, 42 of the 43 eyes had baseline CDVA of logMAR 0.6 or better), and given the “ceiling effect” previously reported [24, 25, 35], it is perhaps unsurprising that we did not observe statistically significant improvement in CDVA in our study, especially in light of the short period of follow-up.

A review [36] has concluded that CS is an important measure of visual function in patients with AMD, based on studies that have shown that, when compared with visual acuity, CS better relates to the ability to perform tasks accurately and efficiently, to discriminate between objects [37] and to judge distances [38]. Also, GD is a clinically important problem in AMD and impacts adversely on mobility performance [39]. In this study, there were observed improvements in CS and GD, for a range of spatial frequencies, under both mesopic and photopic conditions, for the entire study group and for Subgroup A.

A progressive improvement of ROS in response to ranibizumab therapy for nv-AMD, as far as 24 months following the initiation of treatment and in spite of stabilisation of visual acuity after six months, has been previously demonstrated [13]. In the current study, ROS, within the central 5 degrees and within the central 16 degrees, improved significantly for the study group and for Subgroup A, whereas ROS at fixation improved significantly only when the entire study group was considered. Microperimetry examines the light differential threshold at the retina and depends on the intactness of photoreceptors, whereas visual acuity is a measure of the minimum angle of spatial resolution and depends largely on the transparency of the ocular media [40]. Intuitively, therefore, one would expect that measures of ROS are more appropriate than CDVA when attempting to correlate function and morphological changes at the macula, for conditions such as AMD, which is consistent with the findings of the current study. A recent study explored the relationships between macular thickness, CDVA, and ROS in patients previously treated with ranibizumab for nv-AMD and who were now in the maintenance phase of therapy [41]. In brief, intravitreal ranibizumab was administered if CDVA and/or OCT was indicative of active disease. On this basis, and at the study outset, sixteen of 21 eyes were classified “unstable”, therefore requiring further intravitreal anti-VEGF therapy. The remaining five were deemed “stable” and were not administered any further intravitreal injections during the study. Although eyes in both groups maintained visual acuity over the study period, those in the “unstable” group exhibited no significant change in ROS over the period of investigation. However, eyes that were deemed “stable” did exhibit a statistically significant decrease in mean ROS during the study period, indicating that eyes with stable visual acuity and absence of intravitreal fluid may still exhibit a deterioration of visual function reflected in measures of ROS, which may be an indicator of subclinical CNV activity.

 Given the importance of OCT in the diagnosis and decision-to-treat/decision-to-discontinue treatment in cases of nv-AMD, the relationships between observed changes in MFT and observed changes in the psychophysical parameters over the course of the study were analysed. Although there was a significant correlation between observed changes in MFT and observed changes in CDVA (which, notably, became non-significant with the removal of an outlier) and also with observed changes in GD under mesopic conditions at low and high spatial frequencies, the strongest such association was with observed change in ROS, both at fixation, but more robustly, within the central 5 and 16 degrees of fixation. It appears, therefore, that ROS may have an important, but yet to be fully understood, role to play in the monitoring of patients with nv-AMD undergoing serial intravitreal anti-VEGF therapy. 

For the study group and Subgroup A, a significant improvement in reading speed was observed between baseline and exit visits over the course of the study, but this observed improvement did not correlate with a change in CDVA. Such a disparity has been previously observed by Frennesson et al., who suggested that a change in CDVA does not necessarily relate to a change in near vision [42]. 

In an attempt to achieve best outcomes without overtreating patients with nv-AMD, the posology for intravitreal ranibizumab for this condition has recently been revised (http://www.medicines.ie/medicine/11837/SPC/Lucentis+10mg+ml+Solution+for+Injection/#POSOLOGY). This revision of posology was informed by the evolving body of literature since the publication of the phase III MARINA and ANCHOR trials, where monthly injections were given for a period of two years. In brief, it is now recommended that monthly injections are given until best CDVA is achieved and maintained for three consecutive injections, when interruption of treatment is recommended with monthly monitoring. Where a deterioration in CDVA (defined as a loss of five letters), attributable to activity of nv-AMD, is observed, recommencement of treatment is recommended under the same regime. In light of this revised posology, however, the results of our study strongly suggest that CS-guided or ROS-guided re-treatments are likely to be more sensitive indicators of functional deterioration, and would, therefore, prompt recommencement of treatment at an earlier stage than would a deterioration in CDVA, thereby reducing the risk of irrecoverable loss of central vision. 

## 5. Conclusion

This study has demonstrated improvements in many parameters of visual function in eyes with nv-AMD undergoing monthly intravitreal ranibizumab injections. Outcome measures other than CDVA, such as CS, GD, and ROS, should not only be considered in the design of studies investigating nv-AMD, but also in treatment and retreatment strategies for patients with the condition.

## Figures and Tables

**Table 1:** Psychophysical data for study eyes in the entire study group and Subgroup A, between baseline and exit study visits.

Variable	Entire group			Subgroup A		
	Baseline	exit	*P* value	Baseline	exit	*P* value
	mean (±sd)	mean (±sd)		mean (±sd)	mean (±sd)	
CDVA	88 (9)	89 (10)	0.480	89 (12)	91 (13)	0.387
logRAD	0.33 (0.20)	0.28 (0.22)	0.032*	0.24 (0.18)	0.19 (0.19)	0.139
Reading speed	85 (28)	103 (46)	0.019*	85 (26)	118 (56)	0.037*
Mean reading speed	136 (36)	146 (42)	0.005**	148 (28)	166 (36)	0.005**
CS (mesopic)^†^						
Frequency (cpd)						
1.5	19.47 (10.1)	30.22 (21.00)	0.003**	20.30 (12.15)	39.43 (26.12)	0.008**
3	28.93 (19.72)	42.02 (34.04)	0.004**	32.47 (24.01)	53.13 (33.87)	0.036*
6	11.26 (9.78)	15.91 (17.32)	0.002**	10.43 (9.04)	10.14 (6.35)	0.070
12	4.09 (1.17)	4.88 (2.27)	<0.005**	3.80 (0.77)	6.27 (3.35)	0.001**
18	1.98 (0.15)	2.37 (1.25)	<0.005**	1.93 (0.26)	3.07 (1.98)	0.001**
CS (photopic)^†^						
Frequency (cpd)						
1.5	21.99 (13.83)	27.27 (18.91)	0.082	19.51 (11.11)	23.32 (14.91)	0.347
3	35.05 (23.68)	47.63 (30.12)	**0.005∗∗**	40.00 (30.72)	61.07 (32.54)	0.020*
6	19.56 (19.28)	28.44 (30.98)	0.001**	16.14 (16.75)	18.43 (15.90)	0.221
12	5.72 (4.00)	10.16 (15.29)	<0.005**	7.40 (6.53)	19.47 (23.08)	0.025*
18	2.49 (2.59)	3.58 (4.01)	<0.005**	2.33 (1.59)	6.53 (5.82)	0.016*
GD (mesopic)^†^						
Frequency (cpd)						
1.5	11.10 (7.88)	18.76 (15.03)	0.002**	12.40 (9.79)	27.87 (17.80)	0.019*
3	16.47 (12.63)	29.28 (23.67)	<0.005**	17.93 (14.80)	42.13 (30.51)	0.001**
6	7.51 (6.02)	11.49 (11.71)	<0.005**	6.00 (0.00)	7.00 (2.58)	0.134
12	4.02 (0.77)	4.70 (2.89)	<0.005**	3.80 (0.77)	6.00 (4.72)	0.001**
18	1.98 (0.15)	2.09 (0.43)	<0.005**	1.93 (0.26)	2.27 (0.70)	<0.005**
GD (photopic)^†^						
Frequency (cpd)						
1.5	19.94 (12.38)	24.50 (14.79)	0.090	19.02 (13.31)	21.54 (13.27)	0.377
3	35.16 (25.82)	47.33 (33.20)	<0.005**	43.13 (30.86)	67.67 (39.65)	0.039*
6	19.04 (19.46)	28.42 (31.20)	0.001**	15.25 (14.22)	18.50 (19.24)	0.069
12	5.84 (5.96)	7.86 (9.21)	<0.005**	7.53 (9.21)	13.80 (13.66)	0.022*
18	2.40 (1.68)	3.63 (4.05)	<0.005**	2.73 (2.63)	6.67 (5.83)	0.021*
Mean ROS (dB)						
Fixation	8.56 (5.91)	10.20 (5.71)	0.026*	8.36 (7.32)	11.64 (6.72)	0.056
Central 5°	9.63 (4.83)	11.18 (4.48)	0.003**	9.27 (6.46)	12.34 (5.17)	0.013*
Central 16°	11.03 (4.49)	12.11 (4.00)	0.005**	10.32 (5.51)	12.55 (4.42)	0.017*
OCT						
MFT (*μ*m)	233 (59)	205 (40)	0.001**	275 (64)	208 (25)	0.002**
MFV (*μ*m^3^)	0.18 (0.05)	0.16 (0.03)	<0.005**	0.22 (0.05)	0.16 (0.02)	0.002**

Abbreviations: reading speed (at best baseline LogRad value); wpm: words per minute; mean reading speed (for range of LogRad values); CS: contrast sensitivity; mesopic: under mesopic conditions; cpd: cycles per degree; photopic: under photopic conditions; GD: glare disability; ROS: retinotopic ocular sensitivity; dB: decibel; OCT: optical coherence tomography; MFT: mean foveal thickness; MFV: mean foveal volume; —: change was not significant.

Note: The *P* values reported are for the paired *t* test (or the corresponding nonparametric test when the data distribution was non-normal).

*Significant improvement at the 0.05 level (2 tailed).

**Significant improvement at the 0.01 level (2 tailed).

^†^The statistical tests were based on log-transformed data.

**Table 2:** Correlations between observed changes in mean foveal thickness and observed changes in other parameters for the entire study group and Subgroup A.

Variable	Entire group		Subgroup A	
	*r *	*P* value	*r *	*P* value
CDVA	−0.311*	**0.042** ^†^	−0.569*	**0.027**
ROS fixation	−0.411**	**0.008**	−0.500	0.069
ROS central 5°	−0.592**	**<0.001**	−0.611*	**0.020**
ROS central 16°	−0.536**	**<0.001**	−0.554*	**0.040**
logRAD	0.182	0.242	0.505	0.055
Reading speed	0.105	0.501	0.537	0.039*
Mean reading speed	−0.259	0.307	0.033	0.908
LogCSmesopic_1.5cpd	−0.122	0.440	0.133	0.651
LogCSmesopic_3cpd	−0.073	0.645	−0.058	0.843
LogCSmesopic_6cpd	−0.091	0.569	0.193	0.508
LogCSmesopic_12cpd	−0.005	0.973	0.459	0.098
LogCSmesopic_18cpd	−0.041	0.797	0.327	0.254
LogCSphotopic_1.5cpd	0.001	0.997	0.066	0.823
LogCSphotopic_3cpd	−0.087	0.586	−0.086	0.770
LogCSphotopic_6cpd	0.018	0.910	−0.022	0.939
LogCSphotopic_12cpd	−0.165	0.296	0.000	0.999
LogCSphotopic_18cpd	−0.147	0.352	0.260	0.369
LogGDmesopic_1.5cpd	−0.334*	**0.031**	−0.167	0.569
LogGDmesopic_3cpd	−0.344*	**0.026**	−0.206	0.481
LogGDmesopic_6cpd	−0.108	0.494	0.018	0.951
LogGDmesopic_12cpd	0.001	0.997	0.418	0.137
LogGDmesopic_18cpd	−0.348*	**0.024**	−0.246	0.397
LogGDphotopic_1.5cpd	0.166	0.294	0.313	0.277
LogGDphotopic_3cpd	0.157	0.322	0.488	0.077
LogGDphotopic_6cpd	−0.065	0.683	−0.125	0.671
LogGDphotopic_12cpd	−0.208	0.187	0.078	0.792
LogGDphotopic_18cpd	−0.220	0.161	0.140	0.634

Abbreviations: CDVA: corrected-distance visual acuity; ROS: retinotopic ocular sensitivity; CS: contrast sensitivity; mesopic/photopic: under mesopic/photopic conditions; cpd: cycles per degree; GD: glare disability.

*Correlation significant at the 0.05 level (2 tailed).

**Correlation significant at the 0.01 level (2 tailed).

^†^Removal of one outlier.
